# Enhanced Conditional GAN for High-Quality Synthetic Tabular Data Generation in Mobile-Based Cardiovascular Healthcare

**DOI:** 10.3390/s24237673

**Published:** 2024-11-30

**Authors:** Malak Alqulaity, Po Yang

**Affiliations:** Department of Computer Science, University of Sheffield, Sheffield S1 4DP, UK; po.yang@sheffield.ac.uk

**Keywords:** tabular data, generative adversarial networks, synthetic data generation, cardiovascular disease, medical informatics, machine learning in healthcare

## Abstract

The generation of synthetic tabular data has emerged as a critical task in various fields, particularly in healthcare, where data privacy concerns limit the availability of real datasets for research and analysis. This paper presents an enhanced Conditional Generative Adversarial Network (GAN) architecture designed for generating high-quality synthetic tabular data, with a focus on cardiovascular disease datasets that encompass mixed data types and complex feature relationships. The proposed architecture employs specialized sub-networks to process continuous and categorical variables separately, leveraging metadata such as Gaussian Mixture Model (GMM) parameters for continuous attributes and embedding layers for categorical features. By integrating these specialized pathways, the generator produces synthetic samples that closely mimic the statistical properties of the real data. Comprehensive experiments were conducted to compare the proposed architecture with two established models: Conditional Tabular GAN (CTGAN) and Tabular Variational AutoEncoder (TVAE). The evaluation utilized metrics such as the Kolmogorov–Smirnov (KS) test for continuous variables, the Jaccard coefficient for categorical variables, and pairwise correlation analyses. Results indicate that the proposed approach attains a mean KS statistic of 0.3900, demonstrating strong overall performance that outperforms CTGAN (0.4803) and is comparable to TVAE (0.3858). Notably, our approach shows lowest KS statistics for key continuous features, such as total cholesterol (KS = 0.0779), weight (KS = 0.0861), and diastolic blood pressure (KS = 0.0957), indicating its effectiveness in closely replicating real data distributions. Additionally, it achieved a Jaccard coefficient of 1.00 for eight out of eleven categorical variables, effectively preserving categorical distributions. These findings indicate that the proposed architecture captures both distributions and dependencies, providing a robust solution in supporting mobile personalized cardiovascular disease prevention systems.

## 1. Introduction

In the healthcare domain, Electronic Health Records (EHRs) are considered as a valuable resource that significantly contributes to research such as disease investigation and preventive healthcare measures, thus helping in advancing medical informatics and healthcare. These records are frequently organised in a tabular format, where each row representing an individual patient and encompasses a broad range of health and demographic details, such as name, address, weight, blood pressure, heart rate, among others. This structure supports a wide variety of data types and allows for efficient management of large volumes of information. The flexibility of tabular data allows it to exhibit diverse properties depending on the specific information being stored and processed. However, using EHR data for machine learning models is challenging due to issues introduced during data collection, such as systematic errors, human factors, or inherent biases [1]. These problems result in missing, imbalanced, or biased data, which introduce additional complexities to the development and validation of predictive models. Machine learning models trained on such data are prone to producing biased classifiers, which can impact both the accuracy and generalizability of the predictive models.

A viable solution to address these limitations is the generation of synthetic data [2]. This technique reduces issues related to missing or biased data, providing a robust foundation for developing reliable predictive models in the medical domain. By replicating the statistical properties of original data, synthetic data effectively captures distributions and relationships within the data, making it a valuable substitute in various applications. It plays a crucial role in medical research, especially when access to real datasets is restricted, and is increasingly used for testing, evaluation, and statistical disclosure control. Generative Adversarial Networks (GANs), introduced in 2014 [3], have become significantly influential in the field of medical informatics. GANs are deep learning models that capture the complex multidimensional distribution of training data. This capability allows these models to produce new synthetic data points that replicate the real data’s distribution. GANs comprise two parts: a generator that produces realistic data, and a discriminator that evaluates whether the data is real or fake. Through their adversarial interaction, the generator learns to mimic the real data’s distribution effectively enough to deceive the discriminator into accepting it as real data, while the discriminator becomes increasingly adept at differentiating accurately between the real and synthetic samples. Although GANs perform well in data generation tasks on homogenous data, such as images, audio and text, generating high quality synthetic tabular data poses significant challenges.

In recent years, mobile sensing-based healthcare systems have emerged as critical tools in personalized medicine, particularly for cardiovascular health. These systems utilize data from wearable sensors, mobile devices, and EHRs to monitor patient health metrics and deliver customized solutions. However, their effectiveness is often limited by the lack of high-quality datasets required for training robust predictive models. Synthetic datasets designed for these applications enable the integration of diverse data sources into sensing-based systems. They play a crucial role in advancing cardiovascular disease management by addressing this limitation. Such applications emphasize the importance of tackling challenges in tabular data synthesis, particularly for capturing diverse distributions and complex feature relationships.

In the context of data synthesis, tabular data presents significant challenges due to its mixture of continuous and categorical data types. Additionally, the complex relationships among columns add to the difficulty. Moreover, Non-Gaussian distributions in tabular data, particularly those with heavy tails, can significantly contribute to the vanishing gradient problem during the training process. These distributions often include extreme values distant from the mean, posing substantial challenges during training. Furthermore, highly imbalanced categorical columns present a significant challenge in the synthesis of tabular data using GAN. These columns often exhibit strong imbalances where most rows belong to one major category, while only a few are represented in one or more minor categories. These minor categories may contain the most relevant information, and their limited representation may result in fewer training opportunities, potentially causing mode dropouts that might not be detected by the discriminator.

While GANs have shown potential in creating synthetic tabular datasets, challenges persist in producing high-quality data, especially for datasets with missing values and mixed data types. In this paper, we propose an enhanced conditional Tabular GAN architecture designed to more accurately model complex data distributions. This is achieved by leveraging Gaussian Mixture Model (GMM) parameters for continuous features and embedding layers for categorical features, enabling the generator to produce synthetic data that closely aligns with the statistical properties of cardiovascular disease datasets. These enhancements to the generator component are expected to support the development of reliable predictive models in cardiovascular healthcare. We demonstrate the effectiveness of our approach by comparing it with state-of-the-art tabular generative models.

## 2. Related Work

There are several models that use GAN for generating tabular data. The medGAN model is amongst the baseline generative models designed for generating synthetic EHRs [4]. In medical records, each column exhibits a distinct distribution, which complicates the training of GANs. Direct modelling approaches often fail to yield satisfactory outcomes. Consequently, medGAN incorporates an autoencoder within the standard GAN framework to transform raw data into a lower-dimensional representation. This modification enables the effective generation of realistic high-dimensional discrete variables, including binary and count features. It is particularly useful for EHR datasets, where each row corresponds to a patient’s record and columns represent various disease codes.

In the medGAN framework, the generator and discriminator operate in distinct spaces. Specifically, the generator produces a latent representation, while the discriminator evaluates raw data. As a result, the generator’s output must pass through a decoder before it is assessed by the discriminator. The generator and discriminator are trained using the traditional loss function applied in vanilla GANs [3]. The evaluation of synthetic data using univariate (dimension-wise statistics) and multivariate (dimension-wise predictions) methods demonstrated promising results when compared to baseline models such as Variational Autoencoder (VAE) and Stacked Restricted Boltzmann Machines [5].

The initial version of medGAN was designed exclusively for generating discrete data, a restriction that persisted in many subsequent iterations of the model. Research enhanced the medGAN model by incorporating nine demographic features across various modalities, effectively preserving the quality of the generated synthetic data [6]. However, this enhancement still did not include support for continuous data types. Additionally, researchers have explored various GAN architectures to enhance the quality of synthetic data (SD), including the application of Boundary Seeking GAN (BGAN) [7] to develop medBGAN [8]. While BGAN is theoretically capable of handling mixed feature types, medBGAN has primarily been assessed using only aggregated discrete data [8].

A related model, medWGAN [9], introduced by the same team, employed a Wasserstein GAN (WGAN) with Gradient Penalty (WGAN-GP) [10]. This adaptation was motivated by the faster convergence and improved coverage of the sample space offered by WGANs [10]. Comparative experiments indicated that medBGAN outperformed both medGAN and medWGAN across all evaluation metrics. Another model, the Realistic Synthetic Data Generation Method (RSDGM) [11], expanded upon the basic medGAN framework. It incorporated lab test codes and enabled the synthesis of mixed feature types. Nonetheless, the training dataset utilised for RSDGM was limited, containing only a small number of instances and dimensions.

HealthGAN [12] builds on medGAN and Wasserstein GAN with gradient penalty (WGAN-GP) to preserve privacy while maintaining data utility. The model employs a transformation approach adapted from the Synthetic Data Vault (SDV) to handle the mixed categorical and continuous. This transformation maps all features to a normalised range of 0 to 1 for synthesis, with the synthetic data subsequently transformed back to its original scale using mappings derived from the real data. Continuous variables are scaled by subtracting the minimum value and dividing by the range (max–min). For categorical variables, categories are ordered by frequency from most to least common, then the range from zero to one is divided into sections according to each category’s cumulative probability. Each category is matched with its corresponding section, and samples are drawn from these sections using a truncated Gaussian distribution. This process is then reversed to map the synthetic data back to its original categories. In a comparison with different models including medGAN, HealthGAN demonstrated higher overall quality.

Variational Encoder Enhancement GAN (VeeGAN) was developed to address challenges related to instability and mode collapse encountered during the training phase [13]. VeeGAN incorporates a reconstructor network, denoted as Rec(·), which operates in conjunction with the generator to restore underrepresented modes. Unlike medGAN, VeeGAN uses a variational principle to mitigate mode collapse by employing both a KL divergence term and an autoencoder-like reconstruction loss for the latent noise vector. The objective function in VeeGAN [13] is shown in Equation (1):

(1) $$O\left(\gamma ,\theta \right)=KL\left[q_{\gamma }\left(x|z\right)p_{0}\left(z\right)\|p_{\theta }\left(z|x\right)p\left(x\right)\right]-E\left[\log p_{0}\left(z\right)\right]+E[∥z-F_{\theta }\left(G_{\gamma }\left(z\right)\right){∥}^{2}]$$

where the first term represents the KL divergence between the approximate and true posterior distributions, and the second term measures how well the reconstructor can recover the latent vector *z* from the generated samples. This objective formulation helps VeeGAN avoid mode collapse by enforcing consistency between the generated data and the true data distribution. Additionally, it provides a reconstruction signal that guides both the generator and reconstructor networks. VeeGAN shares similarities with medGAN as both utilise an autoencoding approach for sample reconstruction. Conversely, there is a fundamental difference between the two models because VeeGAN applies variational inference and incorporates a KL divergence loss, whereas medGAN relies solely on autoencoding and cross-entropy loss. VeeGAN has been evaluated on several public databases, where it demonstrated exceptional results, both in enhancing the accuracy of synthesis and in stabilising the model throughout the training process [14]. However, its application is limited to continuous data, and it lacks easy generalizability to discrete and binary types of data [14].

TableGAN, an enhanced extension of the deep convolutional GAN (DCGAN), incorporates an additional classifier into the GAN architecture [15]. In addition to adopting the adversarial loss function from DCGAN, TableGAN introduces two new loss functions—information loss and classification loss—designed to improve the realism of the synthetic data produced by the GAN. The standard GAN loss, which is used in most GAN architectures, includes both the generator and discriminator loss functions. For a generator *G* and discriminator *D*, the adversarial loss [3] is defined as shown in Equation (2):

(2) $$\min_{G}\max_{D}V\left(D,G\right)=E_{x∼p_{\mathrm{data}}\left(x\right)}\left[\log D(x)\right]+E_{z∼p_{z}\left(z\right)}\left[\log(1-D(G(z)))\right]$$

where *x* is data from the real distribution, *z* is the noise vector, and $p_{\mathrm{data}}$ and $p_{z}$ are the distributions of real data and input noise respectively. The information loss, as defined in Equation (3) [15], measures the difference between specific statistical characteristics of real and synthetic records.

(3) $$l_{\mathrm{info}}^{G}=\max\left(0,l_{\mathrm{mean}}-\delta_{\mathrm{mean}}\right)+\max\left(0,l_{\mathrm{sd}}-\delta_{\mathrm{sd}}\right)$$

This loss uses a hinge loss function, implemented through the use of max(·), meaning that no loss is registered until a specific threshold of quality degradation is reached. For the information loss $l_{\mathrm{info}}^{G}$, zero loss is recorded as long as $l_{\mathrm{mean}}$ or $l_{\mathrm{sd}}$ remains below the respective threshold $\delta_{\mathrm{mean}}$ or $\delta_{\mathrm{sd}}$. These thresholds serve as adjustable parameters that influence privacy levels in TableGAN. Smaller values for these parameters lead to less privacy, and make the synthetic dataset more closely resemble the real dataset.

The classification loss [15] measures the discrepancy between the classifier’s predicted label and the synthesized label and is computed for both real and synthetic data. For real data, it measures the mismatch between the actual label and the label predicted after certain features are removed, reflecting the classifier’s ability to accurately predict labels even when subjected to modifications. The classification loss for real data is defined in Equation (4), while the classification loss for synthetic data is shown in Equation (5).

(4) $$L_{\mathrm{class}}^{C}=E\left[\ell (x)-C(\mathrm{remove}(x))\right]\;\mathrm{where}\;x∼p_{\mathrm{data}}\left(x\right)$$

This formula measures the expected value of the absolute difference between the true label ℓ(x) and the classifier’s prediction after the alteration of *x*, across the distribution of real data $p_{\mathrm{data}}$. The C(remove(x)) indicates the classifier’s output when specific features from *x* are removed.

For synthetic data, the loss follows a similar pattern, evaluating the label accuracy on synthetic data generated by the GAN after modifying the input to the classifier. The classification loss [15] for synthetic data is defined in Equation (5):

(5) $$L_{\mathrm{class}}^{G}=E\left[\ell (G(z))-C(\mathrm{remove}(G(z)))\right]\;\mathrm{where}\;z∼p_{z}\left(z\right)$$

This equation calculates the expected value of the absolute difference between the true label ℓ(G(z)) and the classifier’s prediction after modifying the synthetic data G(z), across the distribution of noise $p_{z}$. However, because TableGAN implements min-max normalisation across all variables, it encounters a limitation in accurately modelling columns with complex multimodal Gaussian distributions [16].

Tabular Generative Adversarial Network (TGAN), is model specifically designed to generate synthetic tabular data using GANs [17]. This model employs a Long Short-Term Memory (LSTM) network architecture in the generator. This sequential modeling approach captures dependencies between columns by treating each row of the tabular data as a sequence of features. To handle continuous variables with multimodal distributions, TGAN uses a technique called mode-specific normalization. This method identifies the different modes within a continuous feature by clustering them using a Gaussian Mixture Model (GMM) and normalizes the data within each mode separately, preserving the underlying distribution more accurately than standard normalization techniques. For categorical variables, TGAN converts them into one-hot-encoded vectors and adds noise to handle the discrete nature of these variables, enabling the model to process mixed data types seamlessly. The discriminator, on the other hand, is a Multi-Layer Perceptron (MLP) that distinguishes real from generated data. While TGAN effectively handles mixed data types, the use of LSTM networks may increase computational complexity. Additionally, TGAN may not fully address issues related to highly imbalanced categorical variables and missing data.

The Conditional Tabular GAN (CTGAN) represents an advancement over the TGAN model, aiming to preserve the joint distribution across all columns of synthetic data [18], rather than focusing on pairwise correlations as seen in TGAN [17]. CTGAN utilizes a variational Gaussian mixture model (VGM) to transform numerical data, dynamically determining the number of modes for each column. This contrasts with the TGAN model, where the number of modes is predetermined and remains constant across all numerical columns. Moreover, continuous values in CTGAN are represented using a one-hot vector to indicate the mode and a scalar for the value within that mode, while categorical data are one-hot encoded. A significant improvement in CTGAN is its conditional generator, which addresses imbalanced discrete columns by producing synthetic rows based on a given discrete column. This requires a conditional vector to represent specific categorical values. Additionally, the generator loss is modified to enable learning the mapping between the conditional vector and the one-hot-encoded values.

A key approach in CTGAN involves using a sampling technique that ensures the conditional vector is sampled correctly, allowing CTGAN to fully explore all potential values in discrete columns. This technique involves randomly choosing a discrete column and constructing a probability mass function based on the logarithm of its value frequencies. Subsequently, the conditional vector is computed based on this distribution. As a result, techniques such as mode-specific normalization, sampling-based training, and the conditional generator in CTGAN are pivotal for producing high-quality tabular data [18]. Despite these advances, CTGAN sometimes struggles to preserve the categorical dependencies within the data [19]. Therefore, the inability to maintain these dependencies can impact the utility and integrity of the generated datasets. Moreover, CTGAN tends to underperform when working with a limited number of samples [20]. This issue arises because the model may not have enough data to accurately learn the underlying distribution, which can result in lower-quality synthetic data.

Building upon CTGAN, CTAB-GAN enhances the capability to handle mixed data types, including continuous, categorical, and ordinal variables, and addresses challenges such as imbalanced data and complex feature distributions [21]. It incorporates techniques like conditional generation and information loss to improve the fidelity of the generated data. However, CTAB-GAN tends to overrepresent zero values, producing more than are seen in the original data. This indicates that CTAB-GAN could still be improved to better handle cases of extreme imbalance. CTAB-GAN+ further improves the synthesis of tabular data with complex scenarios [16]. This model introduces advanced data transformations and neural network architectures to better capture intricate data patterns, such as skewed distributions and multimodal features. CTAB-GAN+ exhibits superior performance in generating high-quality synthetic data that closely replicates the statistical characteristics of real datasets.

A study presents an Electronic Medical Record WGAN (EMR-WGAN) framework [22]. In this model, the authors use the basic structure of the original GAN but eliminate the autoencoder, arguing that it reduces synthetic data realism by introducing additional noise and lowering performance. The study also introduced a conditional training strategy, integrating concept labels of records within both the generator and discriminator. Additionally, the model adopts Wasserstein divergence and normalization techniques to improve training stability and utility. Moreover, utility measures including latent space representation and first-order proximity are introduced to better capture the underlying data structure and relationships. The study provided a comprehensive comparison of the proposed model, yielded superior performance across realism metrics. However, a limitation of the model is its focus solely on binary features.

Recent research introduces Anonymization Through Data Synthesis Using Generative Adversarial Networks (ADS-GAN) [23]. This approach aims to enable data sharing by generating synthetic datasets that closely match the joint distribution of original EHR data while ensuring privacy through reduced identifiability. The model builds on WGAN-GP and conditional GAN architectures, introducing an innovative ‘identifiability’ metric within the loss function to quantify and control similarity between synthetic and original data. This metric enables setting an identifiability threshold to balance data utility and privacy by maintaining a minimum distance between synthetic and real records. Embedding this identifiability constraint within the generative model’s loss function ensures adequate separation between synthetic and original data, thereby protecting against membership and attribute disclosures. ADS-GAN was evaluated against various benchmark models, including medGAN and WGAN-GP, across four real-world datasets. The findings showed that ADS-GAN outperformed these benchmarks in preserving statistical and correlational structures under privacy constraints. Moreover, utility evaluations demonstrated that synthetic data generated by ADS-GAN offer predictive performance comparable to real data in downstream machine learning tasks.

The Tabular Variational Autoencoder (TVAE) is a generative model designed for producing tabular data [24]. It extends the traditional Variational Autoencoder (VAE) architecture to handle the challenges associated with tabular datasets, such as mixed data types and complex feature relationships. VAEs consist of two main components which are an encoder and a decoder. The encoder maps input data to a latent space, producing a distribution (mean and variance) rather than a single point. The decoder reconstructs the data from samples drawn from this latent distribution. A significant innovation in the TVAE model lies in the design of the output layer in the decoder network. This layer is designed to generate a joint distribution of continuous variables and categorical variables. This design choice is vital for effectively representing the complexities of mixed data types within tabular datasets. TVAE is trained by maximizing the Evidence Lower Bound (ELBO), which provides a lower bound on the log-likelihood of the observed data, guiding the model to learn an accurate representation of the data distribution [24].

## 3. Architecture of a Tabular Generative Adversarial Network

**Figure 1:**
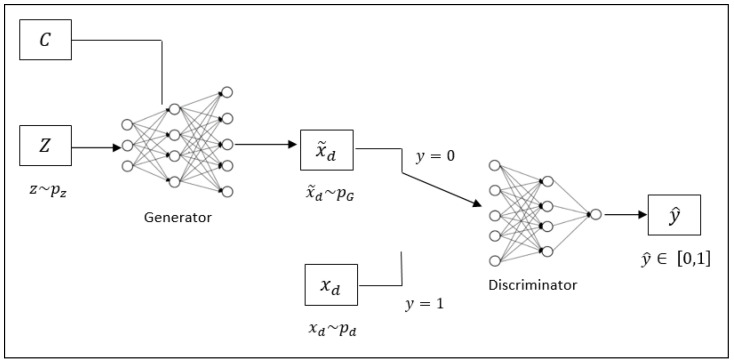
Conditional GAN.

In our approach to creating synthetic tabular data, we build a Conditional GAN, as shown in Figure 1, which is inspired by CTGAN [18]. This architecture consists of two main components: a generator, which receives a noise vector *z*∼$p_{z}$ and a condition vector *C*, and a discriminator, which evaluates whether the generated data ${\tilde{x}}_{d}$ is real or synthetic. The generator learns the distribution of the real dataset $x_{d}$∼$p_{d}$ and produces synthetic data ${\tilde{x}}_{d}$∼$p_{G}$. The discriminator assigns a label *y* to each input to indicate whether the data is real or generated: y=1 for real data $x_{d}$ and y=0 for generated data ${\tilde{x}}_{d}$. The objective is to reduce the difference between the distributions of the real and synthetic data.

The generator in our architecture, referred to as the synthesizer *S*, is specifically trained on existing tabular data *T* to generate synthetic data $\tilde{T}$ that retains the statistical properties of the original table *T*. The synthesizer *S* produces $\tilde{T}$, consisting of *n* rows and *m* columns, where *m* is the sum of *k* continuous and *l* categorical columns. Each column corresponds to a random variable $X_{d,j}$, following the original data’s joint probability distribution $p_{d}$. During training, the synthesizer builds a model $p_{G}$, aiming to match $p_{G}$ with $p_{d}$. Once trained, synthetic data $\tilde{T}$ is generated by sampling from $x_{d}∼p_{G}$, and its quality is assessed using metrics for both continuous and categorical columns.

### 3.1. Data Transformation

The objective of this step is to prepare the data for processing by the generator and discriminator. This typically involves normalizing numerical columns to ensure they fall within similar ranges, commonly either [0, 1] or [−1,1], and encoding categorical columns in a format suitable for GANs. For numerical columns, as mentioned before, these attributes may include multiple modes and follow non-Gaussian distributions. In CTGAN, a Variational Gaussian Mixture Model (VGM) is used to model continuous variables. The VGM applies variational inference to estimate the parameters of each mixture component and the probabilities of each mode, dynamically determining the number of modes based on the complexity of the data. This allows CTGAN to efficiently handle multimodal and non-Gaussian continuous distributions, with each value in a continuous feature represented by a one-hot vector indicating the sampled mode and a scalar normalized to the range of that mode.

In contrast, our approach employs a Bayesian Gaussian Mixture Model (GMM), which uses Bayesian inference to dynamically adapt the number of mixture components based on the data. While variational inference as used in CTGAN is computationally efficient, it may trade some accuracy for speed due to the approximations involved. On the other hand, Bayesian inference provides more comprehensive estimate of the posterior distribution, which can be particularly valuable for capturing uncertainty and modeling datasets with complex or multimodal distributions. This allows our architecture to more effectively adapt to the data by dynamically determining the optimal number of components, potentially leading to more accurate modeling of datasets with varying levels of multimodality. Capturing uncertainty can be valuable for complex or critical datasets, like those in healthcare, where understanding confidence in predictions can improve the reliability of synthetic data and its applications.

The Bayesian GMM [25] clusters each continuous data point into multiple Gaussian distributions, representing the distribution of $C_{i}$ as a weighted sum of *n* Gaussian components. For each component, this model calculates the means $\left(\mu_{1},\ldots ,\mu_{n}\right)$, standard deviations $\left(\sigma_{1},\ldots ,\sigma_{n}\right)$, and the weights of each Gaussian component. For each observation $c_{i,j}$ in the variable $C_{i}$, the probability of originating from each Gaussian component is calculated. The value $c_{i,j}$ is then standardized using the mean and standard deviation of the Gaussian component that has the highest likelihood of generating $c_{i,j}$. The normalised value, denoted as $v_{i,j}$, is computed as shown in Equation (6):

(6) $$v_{i,j}=\frac{c_{i,j}-\mu_{i}^{\left(k\right)}}{\sigma_{i}^{\left(k\right)}}$$

where *k* is determined by using $\arg\max_{k}p_{i,j}^{\left(k\right)}$. Then, to maintain numerical stability and prevent extreme outliers, the normalised value is clipped to the range [−0.99,0.99]. At the end, the transformed variables consist of the clipped values and the associated probabilities, providing a new representation of the continuous variables that effectively captures the multimodal attributes of the data. For each continuous variable, the metadata includes the means, standard deviations, and weights of the Gaussian components derived from the Bayesian GMM, along with the total number of modes employed. This detailed metadata provides an understanding of the underlying distribution characteristics of each variable, which is important for the generator to accurately generate and reconstruct data.

For the categorical transformation, one hot encoder is used to transform the categorical values into a one-hot encoded matrix. This process converts categorical variable values into a form that can be provided directly to the neural networks, which require numerical input. The metadata for each categorical column includes the number of classes (distinct categories) and the original categories. By handling continuous and categorical variables separately, the model ensures that the distinctive characteristics of each variable type are addressed appropriately.

### 3.2. Conditional Generator

**Figure 2:**
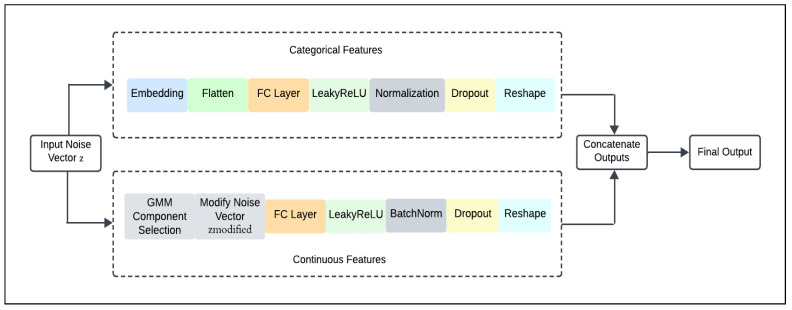
Overall structure of the conditional generator.

As shown in Figure 2, the generator processes continuous and categorical data through separate pathways before concatenating them into the final output. We focus on guiding the generative process to produce synthetic samples that reflect specific patterns within the real data distribution. This is achieved through a conditional architecture where the generator is informed by metadata that specifies the desired mode of generation. This conditioning is essential as it allows the generator to produce samples that are not just random but also represent distinct segments of the data distribution, accurately reflecting their underlying characteristics. A specialised sub-network is constructed to process continuous attributes. This network incorporates metadata about the mean, standard deviation, and weight of each GMM component, derived from our preliminary data analysis. During each generation cycle, the network selects a GMM component depending on the metadata-provided weights, and modifies the input noise vector (z) accordingly. Then, this adjusted noise vector is transformed through a sequence of dense layers, LeakyReLU activations, batch normalisation, and dropout layers to produce the final continuous attributes of the sample.

For categorical features, an embedding layer is used which transforms each categorical value into a dense vector of a size determined based on the number of classes; this embedding representation helps in capturing the relationships between various categories [26]. Additionally, flatten layer which converts the multidimensional output of embeddings into a flat vector that can be processed by subsequent dense layers. After flattening, the data passes through a fully connected layer with a ReLU activation, followed by a normalization layer and dropout to regularize the model. Another fully connected layer is used to further enhance the feature representation and improve the model’s ability to capture the complexity of categorical data distributions. The continuous and categorical processing streams are designed to handle different data types separately until integration. Each stream processes its respective data independently, transforming the inputs through their layers. To combine the results, the outputs of these layers are reshaped into a consistent format. Then, by concatenating these reshaped outputs along the final dimension, we create an integrated feature set. This concatenated output effectively merges the transformed continuous and categorical data into a single, unified representation.

### 3.3. Discriminator

**Figure 3:**
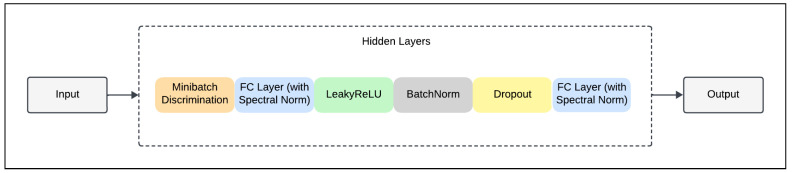
Discriminator framework.

The purpose of the discriminator in the GAN framework is to assess the authenticity of generated samples. This component is crucial for the adversarial training process because it learns to differentiate between real and synthetic data, and help in guiding the generator towards producing increasingly realistic outputs. As illustrated in Figure 3, the discriminator framework consists of several layers. The architecture begins with the Minibatch Discrimination layer [27], which is essential for preventing mode collapse by ensuring that the discriminator can detect diversity within groups of samples, not just evaluate the authenticity of individual samples. It achieves this by computing distances between samples in a transformed feature space, which helps in distinguishing real data from generated counterparts that may lack variety and thus ensures the generator produces diverse outputs. Following the minibatch discrimination, each dense layer within the discriminator is wrapped with Spectral Normalisation. This approach normalises the weight matrices by their largest singular value in order to maintain stability in the training process and prevent the discriminator from overpowering the generator [28].

Moreover, the core of the discriminator’s architecture involves multiple dense layers, each followed by a LeakyReLU activation function. LeakyReLU is chosen because of its ability to allow a small, positive gradient when the unit is inactive and thus aid maintain gradient flow during training. Also, each dense layer is paired with batch normalisation and dropout. The discriminator concludes its processing with a final dense layer that incorporates spectral normalisation but does not employ an activation function. Since there is no activation function at the last dense layer, the network can output a single scalar value. This scalar provides a continuous assessment of authenticity, with higher values suggesting that the input is more likely to be synthetic, while lower values indicate a higher probability that the input is real.

### 3.4. The Loss Function

For the generator to generate realistic samples, the discriminator needs to be trained to maximize the probability of accurately classifying each sample, specifically assigning y=1 to real samples $x_{d}$ and y=0 to generated samples ${\tilde{x}}_{d}$. On the other hand, the generator is trained in an adversarial manner to minimize the likelihood that the discriminator correctly identifies the generated samples as fake, i.e., y=0. This process, as introduced by Goodfellow et al. (2014) [3], wherein the generator and discriminator interact in a minimax game, is represented by Equation (7).

(7) $$\min_{G}\max_{D}V\left(G,D\right)$$

During training, both the generator and discriminator aim to reduce their respective loss functions. Equation (8) shows the loss function for the discriminator, which seeks to maximize the value function V(G,D).

(8) $$l_{D}=-V\left(G,D\right).$$

The loss function for the generator, assuming the discriminator is optimal, is expressed in Equation (9).

(9) $$l_{G}=-l_{D}=V\left(G,D\right).$$

Binary cross-entropy [29], given in Equation (10), is a common loss function that is employed in binary classification tasks, including the training of neural networks in GAN. This function calculates the dissimilarity between predicted probabilities and true binary labels.

(10) BCE=−[y·log(p)+(1−y)·log(1−p)]

where *y* is the true binary label which can either be 0 or 1, *p* is the predicted probability of the positive class, which is label 1. In this function, the term y·log(p) adds the loss in the case of y=1, whereas the term (1−y)·log(1−p) adds the loss when *y* is 0.

The discriminator’s role is maximizing its capability to differentiate between real and generated samples by minimizing the binary cross-entropy loss. This involves assigning high probabilities which are close to 1 to real samples and low probabilities, close to 0, to fake samples. Conversely, the generator seeks to deceive the discriminator by generating samples that are classified as real, thereby increasing the discriminator’s loss. At the end this dynamic interaction leads to improved performance for both models during the training process. However, binary cross-entropy loss can cause training instability, particularly when the discriminator becomes too confident in its predictions, leading to saturation [30]. This saturation can hinder the learning process for both the generator and the discriminator, thereby limiting their ability to improve.

In the context of GAN, the Wasserstein loss is employed as a metric to guide the training process [31]. This approach allows the generator to create samples that more closely resemble the real data distribution. Unlike traditional GANs that use binary cross-entropy loss, Wasserstein GANs offer a more stable training process and can generate higher quality data samples. The Wasserstein loss [31], as defined in Equation (11), works by computing distance between the generated and real data distributions. In practice, this loss is calculated as the maximum difference between the expected value of the discriminator function, which is represented as D(x), applied to both the real and generated data distributions.

(11) $$W\left(P_{d},P_{g}\right)=\max\left(E\left[D\left(x\right)\right]\right)-\min\left(E\left[D\left(G\left(z\right)\right)\right]\right)$$

where E[] denotes the expectation, which is computed over the corresponding distribution. D(x) is the output of the discriminator for a real data sample *x*, while G(z) is the generated data sample produced by the generator from random noise *z*. The advantages of this loss are that it does not max out even when the supports (the underlying data structures) of the generated probability distribution $p_{g}$ and the real data distribution $p_{d}$ exist on low-dimensional manifolds. This allows the discriminator to be trained extensively, possibly even to or near its optimal performance, without causing the issue of vanishing gradients.

This issue arises during neural network training when gradient values become so small that they can no longer effectively adjust the network’s weights during backpropagation process [29]. In the context of GANs, it is vital to avoid vanishing gradients to ensure the generator receives beneficial feedback on its performance, which helps the generator continuously improve its output, making it more closely resemble the real data distribution. Therefore, in our research, we are using Wasserstein loss rather than binary cross-entropy to leverage its benefits in dealing the vanishing gradient issue and improving the generator’s ability to produce outputs that closely resemble the real data distribution.

**Figure 4:**
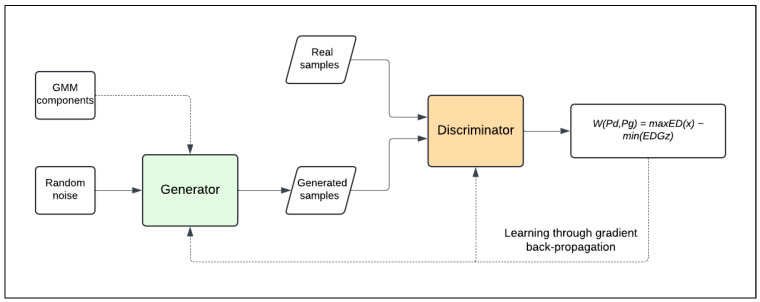
Training process.

In our work, training is performed via gradient back-propagation, as illustrated in Figure 4. This process follows the adversarial framework introduced by Goodfellow et al. (2014) [3], where the generator and discriminator are trained in a minimax game to enhance their respective performance. The first step is called the forward pass, where input data is passed through the network, undergoing linear transformations and non-linear activations to produce the final prediction or classification. Next, the model calculates the loss by comparing the predicted output with the true target values, quantifying the discrepancy between them. Gradients of the loss function are then computed with respect to each weight in the network, indicating how small changes in the weights will affect the overall loss. In the backward pass, these gradients are propagated back through the network, starting from the output layer and moving to the input layer. During this step, the gradients are used to adjust the weights, guided by the Adam optimization algorithm.

The weights are updated in a way that reduces the loss, usually scaled by a learning rate. This process is repeated over multiple epochs until the network converges to a group of weights that minimise the loss function. Efficient training using gradient back-propagation requires attention to several factors. Learning rate which is a critical hyperparameter that controls the step size taken in the weight space. Note that a too high learning rate can cause oscillations and divergence, while a too low learning rate can lead to slow convergence. Additionally, batch size represents the number of samples processed before adjusting the weights. Larger batch sizes provide more accurate gradient estimates but need more memory and computational resources. Furthermore, appropriate weight initialization can significantly affect the convergence speed and overall performance of the network.

## 4. Experimental Results and Analysis

In order to evaluate the performance of our proposed approach in producing synthetic tabular data that closely resembles real datasets, we performed an extensive experimental comparison with both CTGAN and TVAE. The comparison assessed the ability to generate both continuous and categorical data, using several metrics. For continuous columns, we applied a two-sample Kolmogorov–Smirnov (KS) test [32]. This test calculates the maximum difference between the cumulative distribution functions of real and generated datasets, as shown in Equation (12). The KS statistic ranges between 0 and 1, where a lower value suggests a higher level of similarity between the distributions.

(12) $$D_{n,m}=\max\left|F1\left(x\right)-F2\left(y\right)\right|$$

Here, *D* represents the KS statistic, *n* and *m* are the sample sizes of the two datasets, and F1(x) and F2(y) are the respective CDFs.

To assess the categorical data, we utilized the Jaccard coefficient to quantify the similarity between the real and generated data for each categorical column [33]. The Jaccard coefficient, shown in Equation (13), ranges from 0 to 1, where 1 indicates complete similarity.

(13) $$J\left(A,B\right)=\frac{\left|A\cap B\right|}{\left|A\cup B\right|}$$

In this formula, |A∩B| indicates the cardinality of shared observations across both datasets, while |A∪B| is the total number of distinct observations when the two datasets are combined. These metrics help evaluate how effectively the synthetic data replicates the original in both continuous and categorical variables.

In this study, the dataset was derived from the King Faisal Specialist Hospital & Research Centre in Saudi Arabia, comprising 218 patient records. It includes both categorical and continuous data, covering various clinical and demographic features such as age, weight (WT), height (HT), and body mass index (BMI). The categorical features encompass clinical diagnoses and conditions, including estimated glomerular filtration rate (eGFR), prior stroke (P.STRK), prior myocardial infarction (P.MI), atrial fibrillation (A.F), gender, and coronary artery disease history (CAD.prior), among others. Continuous features consist of baseline and follow-up measurements for clinical parameters like Hemoglobin A1c (hA1c), diastolic blood pressure (DBP), low-density lipoprotein cholesterol (LDL), and total cholesterol (T.C.). For example, hA1c (#1), DBP (#1), LDL (#1), and T.C. (#1) were recorded at baseline, while follow-up measurements were taken for the same parameters, along with the coronary artery calcium score (CACS#2.scor). Importantly, the dataset does not contain confidential information such as names, addresses, or ID numbers. All patients in the dataset were over 30 years of age, with males accounting for 76.6% of the population.

Table 1 presents KS statistics for various features across the three models: CTGAN, TVAE, and our proposed approach. The KS statistic measures the maximum difference between the cumulative distribution functions of the real and synthetic data for each feature, where a lower KS value indicates a closer alignment between the synthetic and real data distributions. Our proposed architecture demonstrates strong performance, achieving lower KS statistics for several key features, such as hA1c.#1 (0.2089) and DBP.#1 (0.0957), compared to TVAE (0.2669 and 0.3525, respectively) and CTGAN (0.4355 and 0.3137, respectively). These results suggest that our approach more effectively captures the distributions of these variables, reflecting its strong capability in modeling the underlying data patterns.

Also, it demonstrates superior performance for features like LDL.#1 and T.C.#1, achieving KS statistics of 0.1420 and 0.0779, respectively, which are significantly lower than TVAE (0.3946 and 0.1826) and CTGAN (0.4052 and 0.2786). These findings highlight the ability to accurately replicate the distributions of these clinical measurements. Regarding demographic features such as age, WT, HT, and BMI, our approach again shows strong performance. Notably, the KS statistic for BMI is significantly lower at 0.1488, compared to TVAE at 0.2985 and CTGAN at 0.4580. For Age, our approach achieves a KS statistic of 0.1673, outperforming TVAE (0.3621) and CTGAN (0.2526). For WT, a KS statistic of 0.0861 is recorded, outperforming both TVAE (0.2873) and CTGAN (0.1593). These results highlight the effectiveness of our approach in capturing the distributions of demographic characteristics.

Conversely, for certain features, TVAE achieves lower KS statistics than our model. For example, in the case of CACS#2.scor, TVAE records a KS statistic of 0.5399, which is lower than both our approach (0.7179) and CTGAN (0.8039). Similarly, for LDL.#2, TVAE achieves a KS statistic of 0.4982, outperforming our method (0.9345) and CTGAN (0.7375). For hA1c.#2 and T.C.#2, TVAE also demonstrates superior performance, achieving KS statistics of 0.5142 for both features, compared to 0.9341 for hA1c.#2 and 0.9150 for T.C.#2 in our method. These results suggest that TVAE may be more adept at capturing the distributions of these particular features, potentially due to its variational framework, which can better model complex data structures. The mean KS statistics provide an overall indicator of each model’s performance. We achieve a mean KS of 0.3900, which is comparable to TVAE’s 0.3858 and significantly lower than CTGAN’s 0.4803. This overall performance suggests that both our approach and TVAE are more effective than CTGAN at capturing real data distributions across the evaluated features. The slight difference in mean KS statistics between our approach and TVAE indicates comparable overall performance, with each method demonstrating strengths in different features.

**Figure 5:**
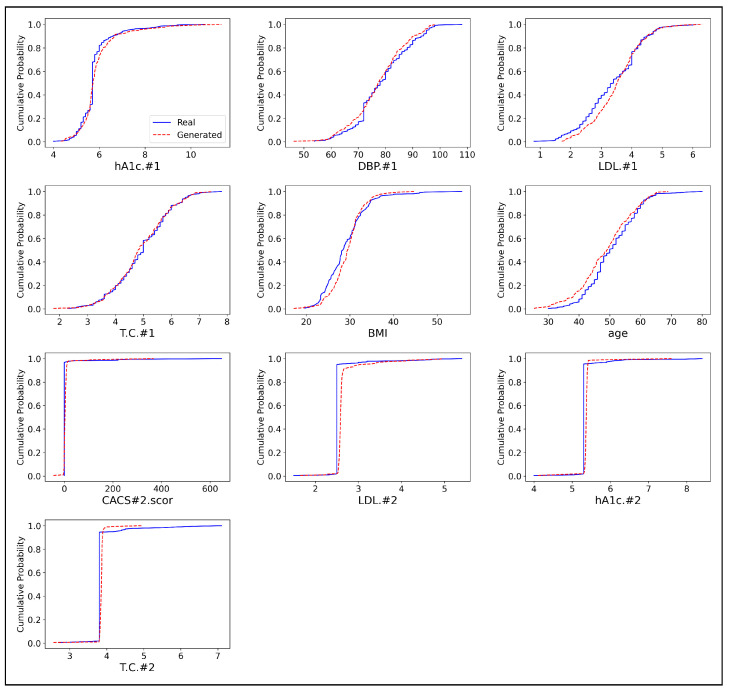
Comparison of Cumulative Distribution Functions (CDFs) for Real and Generated Continuous Data in our approach.

**Figure 6:**
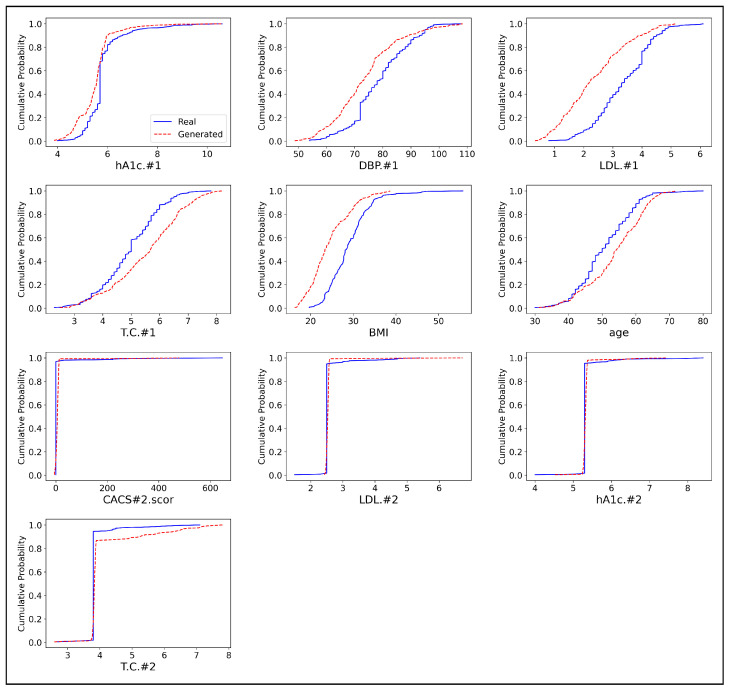
Comparison of Cumulative Distribution Functions (CDFs) for Real and Generated Continuous Data in CTGAN.

**Figure 7:**
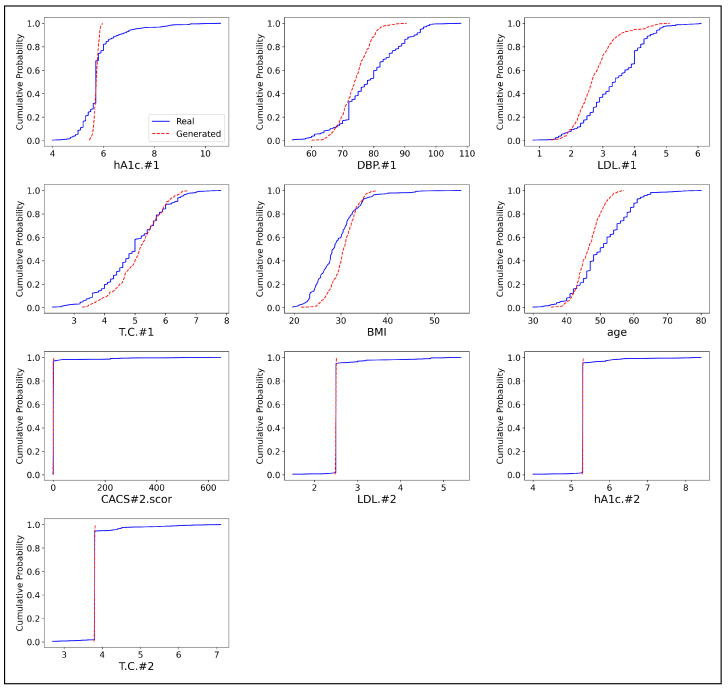
Comparison of Cumulative Distribution Functions (CDFs) for Real and Generated Continuous Data in TVAE.

To further illustrate the models’ performance, we include the cumulative distribution function (CDF) plots for selected features, as shown in Figure 5, Figure 6 and Figure 7. Each plot compares the real and synthetic data distributions generated by CTGAN, TVAE, and our proposed approach, with the x-axis representing the variable values and the y-axis the cumulative probabilities. The red dashed line represents the synthetic data, while the blue solid line represents the real data. A closer alignment between the CDF curves of the real and generated data suggests that the model is more effective in replicating the distribution of the real data. For hA1c.1 and DBP.1, the CDF plots indicate that the synthetic data generated by our approach (Figure 5) closely mirrors the distribution of the real data, consistent with the low KS statistics for these features. In contrast, CTGAN (Figure 6) and TVAE (Figure 7) display larger deviations from the real data, as reflected by their higher KS values. This trend is also observed for LDL.#1 and T.C.#1, where our approach demonstrates a stronger alignment with the real data compared to the other models.

For demographic features such as BMI and age, the method (Figure 5) demonstrates superior performance. The CDF plots show that the synthetic data generated by this approach closely matches the real data distributions, while both CTGAN (Figure 6) and TVAE (Figure 7) exhibit more pronounced differences, which are reflected in higher KS statistics for these features. However, for CACS#2.scor and LDL.#2, TVAE’s synthetic data (Figure 7) aligns more closely with the real data, consistent with its lower KS statistics for these features. Although TVAE displays better KS values, visual inspection reveals slight discrepancies in the distribution shapes that the KS statistic might not fully capture. In contrast, CTGAN (Figure 6) shows the largest deviations for these features, which aligns with its higher KS values. For the final features, hA1c.#2 and T.C.#2, our method (Figure 5) demonstrates stronger performance than CTGAN (Figure 6), as shown by both the KS statistics and CDF plot alignment. Although TVAE (Figure 7) shows slightly better alignment for these features, our approach remains competitive. Overall, across most features, this method (Figure 5) outperforms both CTGAN (Figure 6) and TVAE (Figure 7) in replicating real data distributions. While TVAE performs better for certain features, particularly CACS#2.scor and LDL.#2, our method provides more consistent replication of the real data across a wider range of variables.

To further evaluate the models’ ability to preserve pairwise relationships among continuous features, we computed Pearson correlation matrices for both the real and synthetic datasets, as shown in Table 2. The results indicate that TVAE achieved the highest correlation preservation rate at 77.00%, closely followed by our approach at 76.00%. CTGAN had the lowest preservation rate at 74.00%. These findings suggest that all models are capable of maintaining a substantial proportion of the original correlations, with TVAE marginally outperforming our approach in this aspect. However, the small difference between TVAE and this approach indicates that it remains highly competitive in preserving pairwise relationships among continuous features.

**Figure 8:**
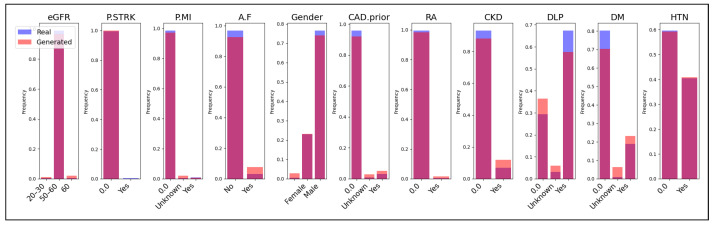
Comparison of Real and Generated Data for Categorical Variables Using Jaccard Coefficient in CTGAN.

**Figure 9:**
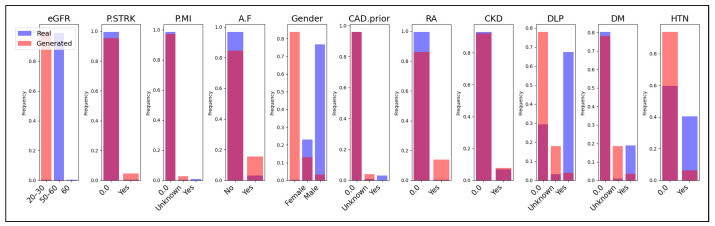
Comparison of Real and Generated Data for Categorical Variables Using Jaccard Coefficient in our approach.

**Figure 10:**
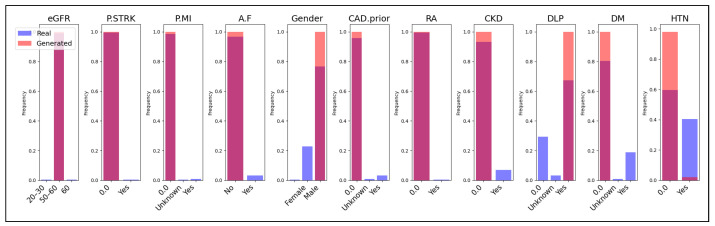
Comparison of Real and Generated Data for Categorical Variables Using Jaccard Coefficient in TVAE.

Additionally, we present a comparison of categorical features between the real and generated data in Figure 8, Figure 9 and Figure 10. Each subplot within these figures represents a different categorical feature, displaying the frequency of each category in both the real (blue bars) and generated (magenta bars) datasets. The Jaccard coefficient provides a quantitative measure of similarity between the real and synthetic categories for each feature. The results in Table 3 and the visual comparisons in Figure 8, Figure 9 and Figure 10 indicate that both CTGAN and our proposed method generally achieve higher Jaccard coefficients compared to TVAE. CTGAN attains a Jaccard coefficient of 1.00 for most features, signifying perfect overlap between the categories in the real and synthetic datasets. Our method achieves high coefficients, with values of 1.00 for eight out of eleven features and 0.67 for three features. In contrast, TVAE exhibits lower Jaccard coefficients across most features, ranging from 0.33 to 0.50, except for HTN, where it achieves 1.00. For features such as A.F, Gender, RA, CKD, DLP, DM, and HTN, both CTGAN and our method achieve a perfect Jaccard coefficient of 1.00, as depicted in Figure 8 and Figure 9. This demonstrates excellent preservation of categorical distributions and indicates that both models effectively capture the categories present in the real data for these critical medical features.

However, differences emerge in features like eGFR, P.MI, and CAD.prior, as shown in Figure 9. While CTGAN maintains a coefficient of 1.00 for these features(see Figure 8), our method records a coefficient of 0.67, which is still notably higher than TVAE’s 0.33 for the same features. The frequency distributions in Figure 9 reveal that our method captures a significant portion of the categories but does not achieve the complete overlap seen with CTGAN. A notable observation is the performance on the P.STRK feature, where our method achieves a perfect coefficient of 1.00, outperforming both CTGAN (0.50) and TVAE (0.50). The visual comparison in Figure 9 highlights the superior ability of our method to replicate the categorical distribution of this feature, likely due to the embedding layers for categorical variables that enhance the capture of category relationships. Overall, TVAE underperforms in preserving categorical distributions, with Jaccard coefficients mostly at 0.33 or 0.50, as shown in Figure 8, Figure 9 and Figure 10. This suggests that TVAE struggles to accurately replicate the category sets present in the real data. This limitation may affect the usefulness of synthetic data generated by TVAE, particularly in analyses that depend significantly on categorical variables.

## 5. Discussion and Potential Applications

The experimental analysis demonstrates that our proposed architecture offers significant advancements in generating synthetic tabular data that closely replicating the real datasets. By comparing our findings with two established generative models—CTGAN and TVAE—we have identified key areas where our enhanced framework excels and others where further refinement is necessary. The proposed approach consistently outperforms the other models in replicating the distributions of several continuous features. Specifically, for features such as hA1c.#1, DBP.#1, LDL.#1, T.C.#1, age, HT, and BMI, our approach achieves the lowest KS values. This indicates a stronger ability to capture the underlying statistical properties of these variables. The integration of Bayesian GMMs allows for dynamic adaptation to the data, capturing multimodal distributions more effectively than the variational inference used in CTGAN. The enhanced performance can also be attributed to the specialized sub-networks designed for each continuous attribute, which incorporate metadata about the mean, standard deviation, and weight of each GMM component. By adjusting the input noise vector based on these components, the generator produces synthetic samples closely aligned with the real data distributions. Furthermore, the architecture employs dense layers, activation functions, batch normalization, and dropout layers to refine the generated features, enhancing the model’s capacity to mimic complex data patterns.

In terms of categorical features, this approach demonstrates competitive performance when compared to CTGAN and outperforms TVAE. The Jaccard coefficients reveal that our approach effectively preserves the category sets for most features, achieving a coefficient of 1.00 for eight out of eleven categorical variables. The embedding layers utilized for categorical data likely contribute to this by capturing relationships between categories, enhancing the generator’s ability to replicate the categorical distributions accurately. Conversely, the higher performance of CTGAN in generating categorical features can be attributed to the use of a conditional vector, which helps the model address the issue of imbalanced categorical columns. This vector enables the model to generate synthetic rows that are conditioned on specific values of a discrete (categorical) column. Additionally, the training-by-sampling technique ensures that the conditional vector is properly sampled by uniformly selecting from all possible values within the discrete columns, thereby accounting for the entire range of categories during training.

Compared to medGAN [4] and its variants (medBGAN [8], medWGAN [9]), our proposed approach enhances the ability to handle mixed data types without relying on an autoencoder, which medGAN uses to manage high-dimensional discrete variables. Although medGAN is effective at generating binary and count features commonly found in EHR datasets, it lacks support for continuous data types. Our approach addresses this limitation by employing specialised sub-networks for both continuous and categorical features. For continuous features, we use Bayesian GMMs to more effectively capture complex, multimodal distributions. VeeGAN [13] incorporates a reconstructor network to mitigate mode collapse, primarily focusing on continuous data. However, it faces challenges in handling discrete and binary data types. Our architecture addresses this limitation by integrating embedding layers for categorical features, enabling the effective generation of mixed data types. TableGAN [15] improves data authenticity by incorporating information and classification loss functions. However, its reliance on min-max normalization for all variables limits its ability to model complex multimodal Gaussian distributions. In contrast, our approach uses Bayesian GMMs for continuous variables, which allow more accurate capture of these complex distributions. This improvement is reflected in lower KS statistics for key continuous features.

Despite its strengths, our approach has limitations in replicating certain features where TVAE demonstrates superior performance. For instance, in features like CACS#2.scor, LDL.#2, hA1c.#2, and T.C.#2, TVAE achieves lower KS statistics, suggesting a closer alignment with the real data distributions for these variables. This may indicate that TVAE’s variational framework is better suited to capturing the complex data structures inherent in these features. The comparatively lower performance of our approach on these variables may stem from the increased complexity and variability associated with them, highlighting the need for further enhancements to better model such complexities. Additionally, while the Bayesian GMM is a robust method for capturing multimodal distributions, it also increases computational complexity, which could limit the scalability of our approach, especially in settings with limited computational resources. Lastly, although the proposed model performed well with this specific dataset, its generalizability to other medical datasets is uncertain and would benefit from further testing to confirm its broader applicability.

High-quality synthetic data is essential for advancing machine learning applications in healthcare, particularly when access to real-world data is restricted due to ethical or regulatory constraints. Mobile sensing-based healthcare systems, which collect data from wearable devices, mobile applications, and electronic health records, have become critical tools in personalized medicine, especially for managing cardiovascular health. These systems rely on real-time data collection and processing to monitor patient health and deliver personalised recommendations. However, they face significant challenges due to the limited availability of high-quality datasets for training machine learning models. The synthetic tabular data generated by the proposed Conditional GAN offers a viable solution by replicating realistic data distributions. This approach supports the development and evaluation of predictive models for mobile health applications. Cardiovascular disease management through mobile sensing systems depends on data from wearable devices and electronic health records, which combine continuous features (e.g., heart rate, blood pressure) and categorical features (e.g., medication status, comorbidities). The proposed architecture’s ability to capture complex feature relationships and maintain statistical properties makes it well-suited for generating synthetic datasets in these contexts. By augmenting limited real-world data, the synthetic datasets improve model performance and adaptability in mobile healthcare applications.

Future research could focus on several strategies to enhance the performance and applicability of our approach. Integrating components that model higher-order dependencies or incorporating elements of variational techniques within GANs may improve the capture of complex data structures. Additionally, exploring alternative GAN architectures could enhance model stability and the quality of generated data. Investigating advanced sampling approaches would help ensure that minority classes are well represented, thereby improving the model’s ability to generate balanced synthetic datasets. Finally, evaluating the model on a diverse range of medical datasets would help assess its robustness and adaptability. Despite its limitations, our approach bridges the gap observed in existing models like CTGAN and TVAE. CTGAN, while strong in preserving categorical distributions with perfect Jaccard coefficients for most features, generally underperforms in replicating continuous data distributions, as indicated by higher KS statistics. TVAE, on the other hand, demonstrates strong performance in replicating continuous features but struggles to achieve high Jaccard coefficients for categorical variables. This approach delivers robust performance in replicating continuous data distributions while maintaining competitive results in handling categorical data. This balance suggests that it is well-suited for generating synthetic datasets where both continuous and categorical variables are crucial.

## 6. Conclusions

This study introduces an enhanced Conditional GAN architecture that enhances the generation of high-quality synthetic tabular data, particularly for cardiovascular disease research. By incorporating specialized sub-networks for continuous and categorical variables and leveraging metadata such as GMM parameters and embedding representations, our approach more effectively captures complex distributions and relationships in mixed data types compared to previous models. Experimental analysis shows that this architecture achieves a mean KS statistic of 0.3900, surpassing CTGAN’s 0.4803 and closely aligns with TVAE’s 0.3858. Notably, it produces low KS values for critical continuous features, such as total cholesterol (KS = 0.0779), weight (KS = 0.0861), diastolic blood pressure (KS = 0.0957), and height (KS = 0.1045), reflecting higher accuracy in replicating real data distributions. It also demonstrates a strong ability to preserve pairwise variable relationships, which is crucial for ensuring the utility of synthetic data in subsequent analytical tasks. For categorical variables, our approach attains a Jaccard coefficient of 1.00 for eight of eleven features, highlighting its effectiveness in preserving categorical data structures. Overall, this architecture represents a significant step forward in synthetic tabular data generation, providing an effective, balanced approach that handles mixed data types and preserves essential data properties. Its ability to generate high-quality synthetic data offers promise for advancing research in medical informatics and other fields, particularly where privacy, scalability, and data availability are critical constraints.

## Figures and Tables

**Table 1:** KS Statistics for Continuous Features Across Models.

Feature	CTGAN [18]	TVAE [24]	Our Approach
hA1c.#1	0.4355	0.2669	**0.2089**
DBP.#1	0.3137	0.3525	**0.0957**
LDL.#1	0.4052	0.3946	**0.1420**
T.C.#1	0.2786	0.1826	**0.0779**
CACS#2.scor	0.8039	**0.5399**	0.7179
hA1c.#2	0.6301	**0.5142**	0.9341
DBP.#2	0.9011	**0.5011**	0.5371
LDL.#2	0.7375	**0.4982**	0.9345
T.C.#2	0.7210	**0.5142**	0.9150
Age	0.2526	0.3621	**0.1673**
WT	0.1593	0.2873	**0.0861**
HT	0.1477	0.3027	**0.1045**
BMI	0.4580	0.2985	**0.1488**
**Mean KS**	0.4803	**0.3858**	0.3900

**Table 2:** Correlation Preservation Rates Across Models.

Model	Correlation Preservation (%)
TVAE [24]	77.00%
Our approach	76.00%
CTGAN [18]	74.00%

**Table 3:** Jaccard Coefficients for Categorical Features Across Models.

Feature	CTGAN [18]	TVAE [24]	Our Approach
eGFR	**1.00**	0.33	0.67
P.STRK	0.50	0.50	**1.00**
P.MI	**1.00**	0.33	0.67
A.F	**1.00**	0.50	**1.00**
Gender	**1.00**	0.33	**1.00**
CAD.prior	**1.00**	0.33	0.67
RA	**1.00**	0.50	**1.00**
CKD	**1.00**	0.33	**1.00**
DLP	**1.00**	0.33	**1.00**
DM	**1.00**	0.33	**1.00**
HTN	**1.00**	**1.00**	**1.00**

## Data Availability

Restrictions apply to the datasets.
